# Teaching handover in undergraduate education: an evidence-based multi-disciplinary approach

**DOI:** 10.15694/mep.2019.000100.1

**Published:** 2019-05-02

**Authors:** Morris Gordon, Ciaran Grafton-Clarke, Elaine Hill

**Affiliations:** 1 University of Central Lancashire; 2 University of Leicester

**Keywords:** Handover, undergraduate medical education, multi-disciplinary

## Abstract

This article was migrated. The article was marked as recommended.

Poor standards of handover threaten patient safety and continuity of care, contributing significantly to morbidity and mortality. Handover practices has risen to the forefront of the patient safety agenda, with a call to develop and implement undergraduate handover modules into undergraduate healthcare education. Recent systematic reviews demonstrate a common failure of educational interventions to demonstrate a theoretical and pedagogical framework underpinning the delivery of education and method of assessment.

The authors developed and piloted a multi-disciplinary evidence-based undergraduate handover training program to health care students studying at a UK university. The intervention was designed based on underpinning educational theories. It has been developed in a manner that supports dissemination and replication, with a model that is cost effective.

The intervention was designed to assess learner reaction, attitudes and confidence, and knowledge and skills. This was achieved through a pre- and post-intervention attitude questionnaire, and an externally validated pre- and post-intervention knowledge assessment.

46 undergraduate students participated, with a statistically significant increase in self-reported attitudes (p < 0.001) and knowledge (p < 0.001) following the handover intervention. Students participated from the disciplines of medicine, adult nursing, pharmacy, mental health nursing, paramedic practice and operating department practioners.

This intervention serves as a significant resource for those looking to develop local interventions and stands as a truly multi-disciplinary approach to handover education, mirroring the clinical reality. The introduction of this handover intervention immediately improves the attitudes, knowledge and skills of undergraduate healthcare students. Future work should sample beyond the selected 6 professions, investigating the transference of outcomes to the workplace, as well as the impact on patient safety.

## Introduction

Clinical handover may be defined as the transfer of professional responsibility and accountability for the care of a patient to another person or professional group on a temporary or permanent basis ( Blyth, Bost, & Shiels, 2017; Kicken, Van der Klink, Barach, & Boshuizen, 2012). The fundamental aim of handover is to achieve the effective communication of high-quality clinical information, enabling inter-professional collaboration, improvements in patient safety; and forms an integral component in the continuity of patient care ( Gordon, Hill, Stojan, & Daniel, 2018). Handover requires both systematic and individual attention, the development of evidence-based education delivery and assessment methodology ( Darbyshire, Gordon, & Baker, 2013; Gordon et al., 2018), implementation and facilitation within the workplace ( National Patient Safety Agency, 2004), and a sustained effort to ensure its importance in preserving patient safety is maintained ( Johnson & Cowin, 2013; Tucker & Fox, 2014).

Poor standards of handover continue to threaten patient safety and continuity of care, primarily through non-transmission and miscommunication of critical information ( World Health Organisation, 2007). This can lead to longer inpatient admissions and reducing levels of satisfaction patients have in their care. Poor quality handovers contribute significantly to morbidity and mortality through delays in assessment and diagnostics, inappropriate treatment regimens, and associated medical errors ( Dekosky, Gangopadhyaya, Chan, & Arora, 2013; Keogh, 2013).The fiscal implications of breakdowns in communication secondary to ineffective handovers has been widely documented ( Donaldson, 2002), and is a major root cause factor in successful negligence claims, superseding inadequate skill levels of practitioners as the leading cause of permanent disability ( Greenberg et al., 2007; Zinn, 1995).

Handover features as the focus of three major reports published by healthcare regulators in the United Kingdom and Australlia, and provide a number of reccommendations govering best practice in handover of care ( Australian Commission on Safety and Quality in Health Care, 2012; Junior Doctors Committee, 2016; Royal College of Physicians, 2013). The High 5s project launched in 2006 by the World Health Organisation aimed to address major concerns regarding patient safety on a global level, of which safe patient handover is integrated within the solutions of important patient safety problems ( World Health Organisation, 2013). In addition to the impact of handover on the quality of patient care, it is important to recognise the implications of sub-optimal handover on the healthcare staff involved, who may feel unsupported, and not in control of the care of their patients; resulting in emotional distress and clinical uncertainty ( Cleland, Ross, Miller, & Patey, 2009).

Legislative and policy strategies to address error resulting from physical and cognitive fatigue in healthcare workers include the implementation of the European Working Time Directive ( Maybury, 2014), which has directly impacted on the number of shift changes involving healthcare professionals, mainly junior medical staff and nurses. This has led to a subsequent increase in the frequency of handover and shift working, disrupting continuity and further amplifying the cumulative impact of sub-optimal handovers. It is important to note that poor handover skills are not characteristic of any particular professional group, and are a premise and responsibility of all healthcare professionals ( Doyle & Cruickshank, 2012). A recent survey of foundation trainee doctors in the UK highlighted a lack of formal handover training during their undergraduate studies ( Horwitz, Krumholz, Green, & Huot, 2006), resulting in the most junior of doctors feeling unprepared to handover often the most critically unwell patients within the hospital environment ( Cleland et al., 2009). A number of leading bodies, such as the Junior Doctor Committee of the British Medical Association (JDC), have called for the development and implementation of undergraduate handover training modules into every medical school curriculum ( Junior Doctors Committee, 2016) as one of many priorities of action to improve patient safety resulting from sub-optimal handover. The lack of evidence-based handover education persists within other healthcare professions, specifically midwifery and operating department practioners ( Gordon & Findley, 2011; Gordon et al., 2018) In the field of nursing, effective handover and information communication skills are not taught formally during academic education; rather, nurses learn such skills during their daily practice from more experienced nurses ( Lally, 1999).

A systematic review, alongside its recent update, investigating the efficacy of educational interventions aiming to improve handover concluded that there is a growing research base stipulating that handover performance can be improved through education ( Gordon & Findley, 2011; Gordon et al., 2018). Importantly, conclusions pertaining to patient safety outcomes and the transfer of skills from the classroom environment to the workplace cannot yet be drawn, mainly due to the lack of scientifically robust long-term retention studies ( Gordon & Findley, 2011; Gordon et al., 2018). A common theme amongst the interventions were a failure to demonstrate a theoretical and pedagogical framework, and a significant variability in the delivery of education and method of assessment, with a resultant inability to disseminate and replicate handover training modules ( Gordon et al., 2018; Manser & Foster, 2011). Furthermore, the recent descent towards web-based, non-contact teaching methods, with the restriction of teaching tools to handover mnemonics and templates, demonstrates the forgo of evidence-based educational methodology with time economics. Crucially, despite the multi-professional nature of patient care, inter-professional handover educational interventions are rare ( Gordon & Findley, 2011; Gordon et al., 2018; Manser & Foster, 2011).

Published undergraduate handover training interventions typically evaluate self-reported attitudes and confidence levels ( Smith, Peterson, & Beck, 2015), rather than improvements in knowledge or performance ( Chu et al., 2010). Finally, the ability to identify the optimal timing for handover training delivery based on previous research is indeterminate, resulting from a failure of intervention description disclosure and a lack of long-term patient safety studies ( Gordon et al., 2018). The updated systematic review showed that despite an increase in the number of published works, there continues to be a paucity of information on content, pedagogy, or underpinning theory; albeit slight improvements were noticed when compared to the original review ( Gordon & Findley, 2011; Gordon et al., 2018).

The authors of this paper developed and piloted a multi-disciplinary evidence-based undergraduate training program. Its pedagogy is grounded in the two previous systematic reviews and associated theoretical constructs. Additionally, in line with the international patient safety discourse, a multidisciplinary package was developed ( World Health Organisation, 2013).

## Methods

We offered a non-compulsory handover workshop to all undergraduate students enrolled at the University of Central Lancashire (UCLan), training to become one of the following: doctor, pharmacist, adult nurse, mental health nurse, paramedic, operating department practitioner (ODP), child nurse or midwife. drawing from multiple schools and faculties. We delivered two identical two-hour handover workshops with 21-25 attendees per session in June 2017, after the university’s summative examination period. The resources required to deliver each workshop include a generic teaching classroom, projected computer facilities with audio output, 2-3 expert facilitators and printed resources for learning and evaluation (Appendix 1 - 5). This two-hour workshop can be delivered to 40 students with 2-3 facilitators, at an estimated cost of £304 per session (using standard university tariff structure, 2 hours for all 3 facilitators, and a total of 6 hours of preparation and administration for the course).

### Education intervention description

The teaching intervention was designed based on key studies identified within the previous systematic reviews ( Gordon & Findley, 2011; Gordon et al., 2018) and an educational model guiding handover education, referred to as the three pillars of handover education ( Darbyshire et al., 2013). Table 1 highlights these, in addition to the underpinning educational theories and implications for handover education.

**Table 1.** Pillars of education, underpinning theory, and implications for handover education ( Darbyshire, Gordon and Baker, 2013; Gordon et al., 2018).

Pillar of Handover Education	Related Theory	Implications for Education
**Pillar 1:** Information management	Egocentric heuristic: *Overestimation of communication skill ability leads to omission / miscommunication of vital information at handover*	Role play and scenarios to develop communication skills: *To encourage improved checking of information transferred and understanding*
**Pillar 2:** Responsibility and accountability	Agency theory: *patients do not have access to the information required to make an accurate judgment upon whether a doctor is behaving in their best interest*	Discussions of error experiences: *To enhance professional responsibility in learners*
**Pillar 3:** Systems to facilitate handover	Coordination cost: *refers to the financial cost and/ or time cost associated with the systems required to manage increasingly complex handovers*	Education of mnemonic devices, handover checklist and systems: *To reduce the risk of error and increase patient safety*

A map of the sessions components in relation to the three pillars of education and Gagne’s nine events of instruction is presented in Table 2 ( Kruse, 2008). Additionally, content was informed from a number of the studies within the systematic review previously described ( Gordon & Findley, 2011; Gordon et al., 2018). The full lesson plan can be found in Appendix 1, and all associated learning resources in Appendix 2 - 5. As illustrated in Table 2, the workshop combined elements of didactic teaching, small-group role-play with skills application, and large group discussions. For verbal handover, we employed the well-recognised SBAR strategy (Situation, Background, Assessment, Recommendation) within the context of these practical activities, with underpinning of the key error awareness and communication theoretical constructs ( Wentworth et al., 2012). A focus was placed on the importance of interactive questioning, and the role of read-back, as emphasied in the SAIF-IR strategy ( Chu et al., 2009). The rationale behind this is that it emphasises the responsibility of both the giver and recipient of handover information to challenge the previously described egocentric heuristic ( Chang, Arora, Lev-Ari, D’Arcy, & Keysar, 2010). An externally validated handover evaluation instrument allowed the students to appraise the quality of the handover they were receiving from their student colleagues ( Farnan et al., 2010) (Supplemental material).

**Table 2.** Session map, Gagne’s nine events and pillar of education ( Darbyshire et al., 2013; Gordon, 2013; Gordon et al., 2018; Smith et al., 2015).

Session Map	Gagne’s Nine Events	Pillar of Education
**Pre-material** (10 minutes): Consent Pre-attitudes questionnaire Pre-knowledge test		
**Introduction** (10 minutes) IcebreakerAims & objectives	2 - describes the goal; learning objectives	
**Human factors as a source of error** (20 minutes) Video scenario Sub-group discussions Group debrief	1 - gains attention	(2) Responsibility & accountability
**Handover information and ‘bad’ example** (15 minutes) Video example of poor handover Discussion of importance of a good handover Introduction of SBAR and associated techniques	1 - gains attention 3 - stimulate the recall of prior knowledge 4 - present material to be learned; 5 - provide guidance for learning	(1) Information management (2) Responsibility & accountability (3) Systems to facilitate handover
**Practice SBAR** Translate document into SBAR format	6 - elicit performance	(1) Information management (3) Systems to facilitate handover
**Practice scenarios** Facilitator live demonstration of a good handover Grouped practice of SBAR handover using templates, performance judged against set criteria (CEX instrument) [Two students doing handover, with a third rating them using CEX]	1 - gains attention 5 - provide guidance for learning 7 & 8 - provide informative feedback and assess performance test	(1) Information management (3) Systems to facilitate handover
**Debrief and summary** Post-attitudes questionnaire Post-knowledge test		

### Assessment and evaluation

The workshop assessment was designed to evaluate different forms of learning outcome as categorised by Kirkpatrick’s hierarchy for educational assessment ( Kirkpatrick, 1967). Level 1 (learner reaction) was examined using two open-ended questions at the post-intervention stage (Appendix 4). For level 2a (attitudes and confidence), the participants completed a pre- and post-intervention attitude questionnaire, consisting of 10 multiple-choice items ( Smith et al., 2015) (Appendix 3). For level 2b (knowledge and skills), the participants completed a pre- and post-intervention knowledge assessment, consisting of four externally validated multiple-choice questions ( Smith et al., 2015) (Appendix 5).

Evaluations and assessments did not include any identifying information and participation was voluntary. All participants reported information detailing their area of study, year of study and gender. Ethical approval from the university ethics board was obtained (approval number STEMH 373).

### Analysis

The scores were compiled from both workshops and were analysed in aggregate, by discipline, year of study and by gender. We compared pre/post-intervention attitudinal ‘yes’/‘no’ responses and multiple-choice knowledge assessment using McNemar’s statistical test. SPSS Statistics were used for all analyses and considered p < 0.05 to be statistically significant.

## Results/Analysis

### Participant characteristics

A total of 46 undergraduate students participated in the two pilot workshops, attitudinal questionnaires and knowledge assessments. 82.6% (38/46) of participants were female, 17.4% (8/46) were male. Participants were recruited from the following six disciplines: adult nursing (17/46 - 37.0%), ODP (9/46 - 19.6%), medicine (8/46 - 17.4%), pharmacy (5/46 - 10.9%), mental health nursing (4/46 - 8.7%) and paramedic practice (3/46 - 6.5%). Students were recruited from different stages of undergraduate training, with 37.0% in year one (17/46), 47.8% in year two (22/46) and 15.2% in year three (7/46).

### Pre- and post-intervention assessment

All those (46/46) who participated in the workshop completed the pre- and post-intervention attitudinal questionnaire and knowledge assessment. There was a statistically significant increase in the number of ‘yes’ responses in nine of the ten attitude questions following the handover education (p < 0.001) ( Table 3) A ‘yes’ response indicates a positive self-reported attitude. Importantly, there was an increase in participants reporting ‘yes’ in response to ‘I can efficiently handover patients’ following the education intervention (pre-intervention: 35%; post-intervention: 96%, p < 0.001) ( Table 3)

There was a statistically significant increase in the performance of participants across three of the four multiple-choice knowledge questions following the handover workshop ( Table 4). A question regarding the critical elements of written handover demonstrated an improvement following education; however, significance was not demonstrated (pre-intervention 72%; post-intervention 74%, p = 0.705). The mean number of correct responses to the multiple-choice questions improved from 1.54 (CI 1.29-1.79) to 2.61 (CI 2.30-2.91) (p < 0.001) following education.

**Table 3.** Attitudinal questionnaire of 46 undergraduate students before-and-after a handover workshop at the University of Central Lancashire, 2017.

Attitude Assessment	Pre - * n (%)	Post - * n (%)	McNemar’s test **	p-value
I know the critical elements of handover education	18 (39)	46 (100)	28.00	< 0.001
I can create effective written handover communication	19 (41)	44 (96)	25.00	< 0.001
I know a standardised method for verbal handover communication	19 (41)	46 (100)	27.00	< 0.001
I can effectively give verbal handover communication	20 (44)	43 (94)	21.16	< 0.001
I can effectively receive verbal handover communication	27 (59)	44 (96)	15.21	< 0.001
I know how to use “read-backs” in verbal handover communication	4 (9)	44 (96)	34.38	< 0.001
I know how to make contingency plans for my patients	10 (22)	28 (61)	14.70	< 0.001
I can efficiently handover patients	16 (35)	44 (96)	16.70	< 0.001
I am comfortable providing cross-cover care for patients	4 (9)	34 (74)	26.47	< 0.001
Handover communication is important for patient safety	45 (98)	46 (100)	1.00	0.317

Statements rated either ‘yes’ or ‘no’.

* Number of students reporting attitude as ‘yes’.

** McNemar’s chi-squared statistic

**Table 4.** Knowledge assessment of 46 undergraduate students before-and-after a handover workshop at the University of Central Lancashire, 2017.

Knowledge Assessment	Pre - n (%)	Post n (%)	McNemar’s test *	p-value
Which of the following are critical elements of written handover content for every patient?	33 (72)	34 (74)	0.14	0.705
Which of the following are critical elements of verbal handover?	6 (13)	17 (37)	9.31	0.002
Which of the following is the most effect mode of handover communication?	27 (59)	37 (80)	6.25	0.012
Which of the following is the best strategy to verify handover information, such as overnight tasks?	5 (11)	32 (70)	25.14	< 0.001

n represents the number of participants answering the question correctly.

* McNemar’s chi-squared statistic

### General feedback

Comments provided by the attendees were very positive. Participants praised the interactive nature of the workshop as extremely beneficial to their learning, and the expertise and enthusiasm of the facilitators were useful in developing a broadened understanding of the importance of handover with regards to patient safety.

## Discussion

The design and delivery of this novel evidence-based educational intervention is grounded in the most complete and up-to-date literature in the field, ensuring key theoretical principles are considered and proven methods and content employed ( Gordon et al., 2018; Smith et al., 2015). The design also considered the logistics for successful role out of such a programme, and in turn cost, which itself guided design, with an indication of cost presented.

This study demonstrates a statistically significant improvement in self-reported confidence, knowledge and skill level of undergraduate healthcare students in performing handover. The development of handover curriculums is expected to grow in importance and urgency, in light of recommendations provided by the Junior Doctor Committee of the British Medical Association (Junior Doctors Committee, 2016), Royal College of Physicians ( Royal College of Physicians, 2013) and government enquiries into patient safety ( Department of Health, 2015; Zinn, 1995). As such, the intervention presented in this paper provides a significant resource for those looking to develop local interventions, with full learning outcomes, resources, slides and supporting materials available on request from the authors in addition to the materials presented.

By building on the previous systematic reviews ( Gordon & Findley, 2011; Gordon et al., 2018), we can confirm this is the first intervention for handover teaching involving a diverse range of undergraduate health care professionals, mirroring the clinical reality of handover practice. It is also noteworthy that the majority of studies investigating handover curricula have focused on learner reactions and changes in self-reported attitudes (Kirkpatrick 1 and 2a), but often failed to evaluate higher-level outcomes such as knowledge and skills (Kirkpatrick 2b), which was investigated in this study through the application of previously validated pre- and post-intervention knowledge assessments ( Smith et al., 2015). It is important to reiterate that handover curricula should be developed as on-going educational and developmental processes, integrated within other themes of healthcare education such as patient safety, professionalism and communication for clinical practice, rather than a stand-alone workshop.

This study had several limitations. Firstly, despite this study extending to other healthcare disciplines outside of medicine, a number of essential members of the multidisciplinary team were not included within the study, including physiotherapy, midwifery, dietetics and social work students. Secondly, as this was an elective workshop, participants may not be representative of the true cohorts; however, all efforts were made to make the workshop as available and inclusive as possible. Thirdly, the sample size included within this review represents a small proportion of the total undergraduate healthcare student population, and thus performing sub-analysis by discipline, year of study or gender would fail to provide any meaningful conclusions. Further work should undertake power calculations to ensure large enough samples are included within analysis as to answer important questions in this area, such as: ‘at what stage in the undergraduate curriculum should handover education be delivered?’ and ‘what are the differences in handover performance between students from different disciplines?’. Furthermore, this study did not investigate skill decay, transferability to the workplace environment, nor long-term effects on patient safety outcomes and indeed these are the greatest limitations to the generalisability of this study.

Future work should therefore seek to mitigate these limitations with larger sampling from multiple contexts, and transference of outcomes to the workplace should be investigated, as well as the impact on patient safety. We have presented much material to support those seeking to replicate our intervention which will allow for such further research. It is also key for authors publishing such work to address any contextual changes made, as these areas of divergence in the intervention will be as informing for readers as the areas of replication.

## Conclusion

The authors developed an educationally evidence-based multi-disciplinary approach to safe handover in healthcare students. It has been developed in a manner that supports dissemination and replication, with a model that is cost effective. The introduction of this handover intervention immediately improves the attitudes, knowledge and skills of undergraduate healthcare students.

## Take Home Messages

Research investigating the utility of handover education is limited to mainly justification studies, with a paucity of information on content, pedagogy and underpinning educational theory. Additionally, interventions fail to demonstrate an interprofessional approach and provide limited resources allowing for dissemination and replication. The authors have developed an evidence-based intervention serving as a core resource for educators teaching undergraduate healthcare students handover. Further work should assess the long-term outcomes and transference of skill to the workplace, relating to handover educational interventions.

## Notes On Contributors

Morris Gordon is a Consultant Paediatrician and a Professor of evidence and systematic review at the University of Central Lancashire. ORCID: https://orcid.org/0000-0002-1216-5158

Ciaran Grafton-Clarke is a Foundation Year 1 Doctor working in the East Midlands. His involvement with this project was when he was working as a summer intern at the University of Central Lancashire. ORCID: https://orcid.org/0000-0002-8537-0806

Elaine Hill is a senior lecturer in the School of Health Sciences at the University of Central Lancashire.

## Appendices

Appendix 1: Lesson Plan

**Table:** 

Session Map	Gagne’s Nine Events	Pillar of Education
**Pre-material** (10 minutes): • Consent • Pre-attitudes questionnaire • Pre-knowledge test		
**Introduction** (10 minutes)Icebreaker Aims & objectives	2 - describes the goal; learning objectives	
**Human factors as a source of error** (20 minutes) • Video scenario from crash of the century (3 groups). • Sub-group discussions (what went wrong? Why did it go wrong? How could it have been prevented?) • Group debrief	1 - gains attention	(2) Responsibility & accountability
**Handover information and ‘bad’ example** (15 minutes) • Video example of poor handover - Salisbury video • Discussion of importance of a good handover • Introduction of SBAR and associated techniques	1 - gains attention 3 - stimulate the recall of prior knowledge 4 - present material to be learned 5 - provide guidance for learning	(1) Information management (2) Responsibility & accountability (3) Systems to facilitate handover
**Practice SBAR** • Translate document into SBAR format • Single A4 sheet per patient, 6 groups and group feedback	6 - elicit performance	(1) Information management (3) Systems to facilitate handover
**Practice scenarios** • Facilitator live demonstration of a good handover • Paired practice of SBAR handover using templates, performance judged against set criteria (CEX instrument)	1 - gains attention 5 - provide guidance for learning 7 - provide informative feedback 8 - assess performance test	(1) Information management (3) Systems to facilitate handover
**Debrief and summary** • Post-attitudes questionnaire • Post-knowledge test		

Appendix 2: Pre- and Post-Intervention Knowledge Assessment Questions

Choose the one best answer for each of the questions below.

**1**. Which of the following are critical elements of written handoff content for *every* patient?

a. Recent or planned procedures

b. Allergies

c. Day-by-day recap of hospital course

d. Most recent laboratory results

e. Both A & B

**2**. Which of the following are critical elements of verbal handover?

a. Sickest patients discussed last

b. Opportunity to ask questions

c. Reviewing all comorbidities

d. Detailed review of admission history and presentation

e. Both A & B

**3**. Which of the following is the most effective mode of handover communication?

a. Phone conversation with electronic handoff template

b. Recorded message

c. Face-to-face

d. Face-to-face with electronic handoff template

e. Recorded message with emailed written handoff

f. Email written handoff

**4**. Which of the following is the best strategy to verify handover information, such as overnight tasks?

a. Using a check-list

b. Read-back

c. Non-verbal acknowledgment (e.g. nodding)

d. Verbal acknowledgment (e.g. saying ‘okay’)

e. Written acknowledgement (e.g. signing-off)

Participant information (please circle all that apply):

**Table:** 

**Course**	Adult Nursing/Child Nursing/Mental Health Nursing/ Medicine/ODP/Paramedic/Pharmacy
**Year of study**	First/Second/Third
**Gender**	Female/Male

Appendix 3: Pre- and Post-Intervention Attitude Questionnaire

Please answer the following questions:

**Table:** 

Question	Yes	No
1. I know the critical elements of handover communication		
2. I can create effective written handover communication		
3. I know a standardized method for verbal handover communication		
4. I can effectively give verbal handover communication		
5. I can effectively receive verbal handover communication		
6. I know how to use “read-backs” in verbal handover communication		
7. I know how to make contingency plans for my patients		
8. I can efficiently handover patients		
9. I am comfortable providing cross-cover care for patients		
10. Handover communication is important for patient safety.		

Participant information (please circle all that apply):

**Table:** 

**Course**	Adult Nursing/Child Nursing/Mental Health Nursing/ Medicine/ODP/Paramedic/Pharmacy
**Year of study**	First/Second/Third
**Gender**	Female/Male

Appendix 4: Post-Intervention Feedback Questionnaire

We would like to offer these workshops to more students. In order for us to make them as effective as possible, we would like to know what helped you to learn.

What aspects of the workshop helped you to learn best? (Please rate using 1 = helpful, 0= not helpful, N = neutral)

**Table:** 

Aspect	Rating
The room and facilities	
Observing good and bad examples of handover	
Group work exercise using the air crash video	
Having the SBAR mnemonic as a structure	
Explanation of the SBAR mnemonic and how to use it	
Practice at organising written information for handover using SBAR	
Interactive handover practice in pairs	
Opportunity to give feedback on colleagues’ handovers	
Receiving feedback from colleagues on your own handovers	
The opportunity to work in multidisciplinary groups with students from other disciplines	

Thinking of the aspects which helped you to learn, which one helped you the most?

Are there any other comments that you would like to add?

Participant information (please circle all that apply):

**Table:** 

**Course**	Adult Nursing/Child Nursing/Mental Health Nursing/ Medicine/ODP/Paramedic/Pharmacy
**Year of study**	First/Second/Third
**Gender**	Female/Male

Appendix 5: Pre- and Post-Intervention Knowledge Questions

Choose the one best answer for each of the questions below.

**1**. Which of the following are critical elements of written handoff content for *every* patient?

a. Recent or planned procedures

b. Allergies

c. Day-by-day recap of hospital course

d. Most recent laboratory results

e. Both A & B

**2**. Which of the following are critical elements of verbal handover?

a. Sickest patients discussed last

b. Opportunity to ask questions

c. Reviewing all comorbidities

d. Detailed review of admission history and presentation

e. Both A & B

**3**. Which of the following is the most effective mode of handover communication?

a. Phone conversation with electronic handoff template

b. Recorded message

c. Face-to-face

d. Face-to-face with electronic handoff template

e. Recorded message with emailed written handoff

f. Email written handoff

**4**. Which of the following is the best strategy to verify handover information, such as overnight tasks?

a. Using a check-list

b. Read-back

c. Non-verbal acknowledgment (e.g. nodding)

d. Verbal acknowledgment (e.g. saying ‘okay’)

e. Written acknowledgement (e.g. signing-off)

Participant information (please circle all that apply):

**Table:** 

**Course**	Adult Nursing/Child Nursing/Mental Health Nursing/ Medicine/ODP/Paramedic/Pharmacy
**Year of study**	First/Second/Third
**Gender**	Female/Male

## Declarations

The author has declared that there are no conflicts of interest.

## Ethics Statement

Ethical approval was obtained from the University of Central Lancashire - (approval number STEMH 373).

## External Funding

This article has not had any External Funding
