# Supplementary material for: Teaching handover in undergraduate education: an evidence-based multi-disciplinary approach
Source: MedEdPublish (2016). 2019 May 2;8:100. [Version 1] doi: 10.15694/mep.2019.000100.1 (PMC10711954; doi:10.15694/mep.2019.000100.1)
Supplement: Peer review history

# Reviewer report: Reviewer response for version 1

**DOI:** 10.21956/mep.19816.r29842

**Published:** 2019-06-10

**Authors:** Trevor Gibbs

**Affiliations:** 1 AMEE

This review has been migrated. The reviewer awarded 4 stars out of 5 A well written and researched paper that expands on the important issue of the clinical handover; an issue that exposes poor communication and one that fails in the area of patient safety.The authors research in this area led to a very structured intervention that I hope will demonstrate effectiveness in the future- I appreciated the fact that the authors point to this longer-term evaluation.I strongly recommend this paper to all curriculum developers and those faculty involved in patient safety

Reviewer Expertise:

NA

No decision status is available

# Reviewer report: Reviewer response for version 1

**DOI:** 10.21956/mep.19816.r29843

**Published:** 2019-05-09

**Authors:** Barbara Jennings

**Affiliations:** 1 Norwich Medical School

This review has been migrated. The reviewer awarded 4 stars out of 5 This is a research article that describes the design and pilot analysis of a multi-disciplinary undergraduate training session about Handover.In their introduction, the authors clearly articulate the importance of Handover - to avoid miscommunication, to ensure patient safety, and even for the sake of staff wellbeing; they also present the published evidence to justify their case. The thorough justification of their study, positions Handover as a critically important interprofessional clinical skill, which has clinical and health economic impacts. One of the cited studies is a systematic review that identified a lack of evidence-based educational interventions about Handover in curricula. In the description of their methods, the authors explain the pedagogical principles and evidence-base for their workshop intervention; including a mapping to the Kirkpatrick level for each outcome. They present the questionnaires and tabulate the lesson plan and other documents in the methods or in the supplementary material. The study is well designed, and the authors have thoroughly described their methodology to allow replication.This was a successful pilot-intervention, judging from the data presented about the assessments and survey of student experience. The authors carefully list and describe the limitations of the study in the discussion, including the small participant sample size. However, they do not fully justify the quantitative analysis with respect to the statistical test; multiple testing; or the expected effect size of the intervention on the various parameters measured. Could more detail be added?Minor points for revised manuscript:I noted that the methods included an estimate of the cost, this seems surprisingly low and perhaps the authors should explain how it was calculated (e.g. just hourly rate for facilitators, rather than full economic costings?)The paper is well-structured and was interesting to read. The manuscript is nicely written and has a valuable literature review presented through the citations. However, I think a couple of paragraphs could be a little clearer;Abstract: at the end of paragraph one, there is a sentence that challenges the current undergraduate teaching and assessment. However, the authors should be explicit that it is referring to the topic of Handover, otherwise it is a confusing sentence. The final paragraph of the discussion would also benefit from some editing for the sake of clarity.I strongly recommend this paper to the curricular leads for clinical skills and / or patient safety.

Reviewer Expertise:

NA

No decision status is available
